# Psychological Traces of China's Socio-Economic Reforms in the Ultimatum and Dictator Games

**DOI:** 10.1371/journal.pone.0070769

**Published:** 2013-08-14

**Authors:** Liqi Zhu, Gerd Gigerenzer, Gang Huangfu

**Affiliations:** 1 Key Lab of Behavioral Science, Institute of Psychology, Chinese Academy of Sciences, Beijing, China; 2 Max-Planck Institute for Human Development, Berlin, Germany; 3 Beihang University, Beijing, China; George Mason University/Krasnow Institute for Advanced Study, United States of America

## Abstract

Can traces of rapid socio-economic changes within a society be reflected in experimental games? The post-Mao reforms in China provide a unique natural quasi-experiment to study people from the same society who were raised with radically different values about distribution of wealth and altruistic behavior. We tested whether the size of offers in the ultimatum and dictator games are an increasing function of the number of years Chinese citizens experienced of the Mao era (“planned economy”). For the cohort that lived throughout the entire Mao era, we found that mean offers in the two games were substantially higher than what is typically offered in laboratory studies. These offers were also higher than those of two younger Chinese cohorts. In general, the amount offered decreased with less time spent under Mao, while in the oldest group in which every member spent the same amount of time under Mao, the younger members tended to offer more, suggesting an additional effect of early education under Mao and contradicting the alternative hypothesis that generosity increases with age. These results suggest that some of the observed individual differences in the offers made in experimental games can be traced back to the values of the socio-economic era in which individuals grew up.

## Introduction

Experimental studies on the ultimatum game have consistently reported cultural differences even if they failed to find the selfish tribe that conforms to the textbook representations of Homo economicus [1]–[6]. Yet the causal interpretation of cultural differences often remains unclear because cultures differ on many dimensions. Using a “within-society” design, we take advantage of a natural quasi-experiment conducted within one culture, where an entire society was subjected to radically different political and economic conditions in a relatively short period of time. We ask whether the socio-economic reforms in China, specifically the move away from equal distribution of wealth and altruistic collectivism, have left their traces in the social behavior of Chinese citizens in the ultimatum and dictator games.

The People's Republic of China, founded on October 1, 1949, was under Chairman Mao's ideological leadership from its beginning until Mao's death in 1976. In 1978, China launched its economic reform led by Deng Xiaoping, which has resulted in enormous economic growth accompanied by changes in social values. A main moral principle in the Mao era was equal allocation [7], which meant state-owned productive sources, equal distribution of wealth and welfare, and no difference in workers' socio-economic status. For instance, in 1965, China took the extreme egalitarian step of abolishing all ranks and insignia in the People's Liberation Army to emphasize that generals and soldiers are equal. A second moral principle was altruistic collectivism, that is, to subordinate personal interests to the interests of the people. In Mao's words, a person should–like the communist (“internationalist”) physician Dr. Bethune–be “absolutely selfless,” “more concerned about others than about himself,” [8] and ready to “serve the people” [9]. “Only thus can he be considered a communist” [8]. This value system implied that persons were not encouraged to request more compensation for harder work but to accept whatever they got. The system gradually changed with the socio-economic reforms implemented by Deng, who stated: “Getting rid of poverty is the priority of the country” [10]. Equal allocation gave way to allocation in terms of contribution [11]. For Mao, fairness meant equality (low variance) rather than an absolute level of welfare (mean), whereas for Deng, it meant equal chance and free competition, where the absolute level of common prosperity takes priority to equality [12]. The post-Mao economic revolution allowed differences of income, while trying to avoid large discrepancies between the rich and the poor [13].

The ultimatum game, designed by economist Werner Güth [14] is a simple experimental game in which participants can allocate money in more selfish or egalitarian ways. After its invention, the dictator game was designed to control for strategic behavior in the ultimatum game and to measure truly altruistic behavior. Both games have become prominent because in them, people's behavior systematically deviates from game theoretic predictions. In the ultimatum game, the *proposer* is provisionally endowed with a sum of money, often $10, and is asked to offer a portion, nothing, or all to the *responder.* The responder can accept or reject the proposer's offer. If the responder accepts, each player receives the sum allocated by the proposer; if the responder rejects, neither of them receives anything. Game theory predicts for a one-shot ultimatum game that the proposer will offer the smallest positive amount and that the responder will accept it. For instance, if the proposer has $10 (in $1 units), the prediction is that the proposer will offer $1 to the responder, who will accept it because $1 is better than $0. A cross-cultural meta-analysis of 37 papers with 75 ultimatum game experiments reported mean offers of 40% (*SD* = 5.9), and, on average, a 16% (*SD* = 10.7) rejection rate of offers [15]–[16]. These deviations from the predictions of game theory were interpreted as a sign of fairness or, alternatively, as strategic behavior; that is, the offer is not motivated by pro-social motives but by a purely selfish motive combined with the fear that the responder might respond “irrationally” and reject the offer if it is too low.

The dictator game controls for strategic behavior [17] and is identical to the ultimatum game except in that the receiver cannot reject an offer. Positive offers are thus attributed to pro-social motives. Forsythe, Horowitz, Savin, and Sefton [18] found the mean offer in the game to be around 20%, and an analysis of 129 articles published between 1992 and 2009 reported a mean offer of 28% of the pie, with 36% of individuals giving nothing [18]. This suggests that some but not all of the offers in the ultimatum game are strategic; the others appear to be motivated by concerns of fairness.

Does Mao's egalitarian doctrine of serving the people, with its emphasis on unselfishness, equal sharing, and small variance between people, have anything to do with the behavior of Chinese citizens today when they play the ultimatum and dictator games? Would the willingness to share 50–50 differ between people who lived in the Mao era and those who grew up under the post-Mao socio-economic changes? It is by no means evident that the social behavior observed in the small world of experimental games reflects behavior in the “real” world, and apart from studies in small-scale societies [4] , no studies to our knowledge have demonstrated this. To answer these questions, we use the natural experiment in China that divided its recent history into three phases, marked by the beginning and end of the Mao era. We distinguish between three cohorts of Chinese citizens: first, those who were born before 1950; second, the cohort born during Mao's rule, that is, between 1950 and 1976; and third, the post-Mao cohort born after 1976, the time of Mao's death, who grew up entirely under the influence of Deng's new value system. These historical time marks are admittedly very rough divisions and can only be considered as proxies to the different socio-economic value systems that individuals have been taught.

If socio-economic values affect behavior elicited by the ultimatum and dictator games, one should observe differences between Chinese citizens born before Mao's regime (Cohort I), during the regime (Cohort II), and after it (Cohort III). It is far from obvious how to derive predictions of a person's behavior in an experimental game from the socio-economic world in which that person grew up. We decided to formulate and test a simple hypothesis that links behavior to only one variable: the time spent under Mao's regime. For the ultimatum game, the model is:
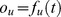
where *o_u_* is the mean offer in the ultimatum game, *f_u_(t)* is a monotonically increasing function, and *t* is the number of years spent under Mao's era (0≤ *t* ≤27). Similarly, for the mean offers *o_d_* in the dictator game, the model predicts:


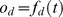
 .Because of the strategic component observed in experiments on the ultimatum game that is absent in the dictator game, we get:
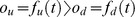



This simple model ignores a multitude of further potential influences that go beyond the mere time spent under Mao, but has the advantage of leading to testable predictions. Every individual in the oldest cohort (born ≤1950) spent the maximum time possible under Mao's regime, that is, about 27 years. These individuals should be the most “equality-motivated” and altruistic. If there is a trace of Mao's socio-economic philosophy in participants' behavior in the games, then the following pattern should be observed:

Prediction 1: 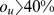 for Cohort I (the oldest cohort), that is, higher than the mean offer typically observed in ultimatum experiments [15], [16]

Prediction 2: 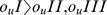 . The mean offer for Cohort I should be higher than the mean offers for each of the other cohorts in the ultimatum game.

Prediction 3: 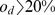
for Cohort I, that is, higher than the mean offer typically observed in dictator experiments [18].

Prediction 4: 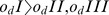. The mean offer for Cohort I should be higher than the mean offers for each of the other cohorts in the dictator game.

Prediction 5: In Cohort II, age and size of offer are positively correlated in both the ultimatum and the dictator game. In Cohorts I and III, by contrast, there should be no positive correlation. This prediction follows directly from the model: Only in the middle cohort does the number of years (*t*) under Mao's regime vary, whereas *t* = maximum for Cohort I and zero for Cohort III. Because the model assumes that *t*, not age, matters, no positive correlation is predicted in Cohorts I and III, where *t* is constant for all members, despite age differences.

Prediction 6: 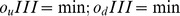. People in Cohort III (born after Mao's death) should be the least equality-motivated and the most self-interested, having spent no time under Mao's regime. They should offer, on average, less money in the ultimatum and dictator games than do the two other cohorts.

As for individuals' behavior as responders in the ultimatum game, the two social values of equal allocation (equality) and altruistic collectivism appear to lead to two different predictions. The political emphasis on equal allocation would suggest that the more years a person has spent under Mao, the higher the minimum accepted offer, that is, the stronger an equal allocation principle is enforced. In contrast, the emphasis on altruistic collectivism and the maxim to accept whatever one gets would suggest the opposite, because what the two players get in total matters more than what one alone gets. Because of this flexibility in deriving predictions, we refrain from making predictions for the responder's behavior and treat this part of the study as a hypothesis-finding experiment.

Studies on the ultimatum and the dictator game have shown that the total amount of money to be distributed and the way participants are compensated can influence behavior [16]. Unaware of any study that has tested this on Chinese citizens, we thus first conducted two pilot studies as a stability check to see whether these factors influence the results.

## Pilot Study 1: Does payment method matter in the ultimatum and dictator games?

There are two ways to compensate the game participants: to pay each one an equal sum, which corresponds to the principle of equal allocation and is standard practice in psychological experiments, or to pay participants depending on their performance, which is the practice in experimental economics. Hertwig and Ortmann [20] and Ortmann and Hertwig [21] reviewed the debate about whether payment method matters and found mixed results. Here, we put the question to an empirical test in the ultimatum and the dictator game.

### Methods

The pilot and main study were approved by the Institutional Review Board of the Institute of Psychology, Chinese Academy of Sciences. Participants gave their written informed consent to take part in the study. Their consent forms and the completed questionnaires were saved separately Data were analyzed anonymously: When data were entered, each participant was given an ID. The consent procedure was approved by the ethics committees.

Two groups of Beijing college students were recruited, with about equal numbers of males and females. In both pilot studies, gender made no difference; thus we report the results independent of gender. One of the groups (*n* = 54) was paid based on performance (according to the decisions they made in the games); the other group (*n* = 57) was paid 20 yuan (Chinese RMB) each for their participation.

Each student took part in both games. The order of the games was counterbalanced between participants, and the instructions were given in written and oral form. Participants were instructed that there were two players in each game, the participant and an anonymous person also familiar with the rules; each would be taking on a different role. In the ultimatum game, the proposer was endowed with 20 yuan and decided what portion of the total sum to allocate to the anonymous second player, the responder. They were also told that if the responder accepted, each player would receive the sum allocated by the proposer; if the responder rejected, neither would receive anything. The participants, who were always in the role of the proposer, were asked to write down the amount they had decided to give to the anonymous person. Once they made a decision, the game was over.

In the dictator game, the players were also allotted 20 yuan. The rules they were given were the same as in the ultimatum game, except that the anonymous responder had to accept the participant's offer.

In the outcome-dependent payment group, participants were told they would receive the remaining amount if their offer was accepted in the ultimatum game and receive the remaining amount unconditionally in the dictator game [22] .In both games, an experimenter served as the anonymous person and accepted any offer except for zero yuan in the ultimatum game. A second experimenter paid participants separately after collecting their answer sheets. In the outcome-independent payment group, each participant was told that he or she would be paid 20 yuan after the entire session for participating in the study. Participants were tested in small groups or individually.

### Results

In both games, offers were slightly higher with outcome-dependent payment, but the standard errors overlapped in both cases (ultimatum game: *p* = .30; dictator game: *p* = .47). The power of the test was .99, .74, and .18 for a large, medium, and small effect size, respectively (*d* = 0.80 large, *d* = 0.50 medium, *d* = 0.20 low). Because payment plan did not appear to influence proposers' offers in both games, we decided to use the simpler outcome-independent payment in the following studies.

## Pilot Study 2: Do stakes affect offers in the ultimatum and dictator games?

In the previous study we used 20-yuan stakes for college students. Since we intended to recruit a diverse sample of adults in the main study, we wanted to raise the stakes to 100 yuan, making it more convenient to calculate percentages. First, however, we checked whether stakes affected the proportion offered in a second pilot study.

### Methods

We recruited another group of 31 college students, comprising 20 males and 11 females. They were tested in both games with stakes of 100 yuan. Like the 57 students in the first pilot study, this group of participants was paid 20 yuan each for their participation. The study procedure was also the same as in pilot study 1.

### Results

In both games, proposers' mean offers were slightly higher in the 20-yuan condition, but the standard errors overlap in both cases (ultimatum game: *p* = .56; dictator game: *p* = .44). The power of the test was .94, .60, and .14 for a large, medium, and small effect size, respectively (*d* = 0.80 large, *d* = 0.50 medium, *d* = 0.20 small). The results indicate that the difference between 20 and 100 yuan has little or no effect on the percentage proposed in both games. This finding is consistent with observations in other cultures that the amount of money at stake has little effect on the offers in the ultimatum game and allocations in the dictator game [23].

## Do cohort differences in the ultimatum and dictator games exist?

Given the results of pilot studies 1 and 2 (see Table 1), for the main study we chose an outcome-independent payment method and stakes of 100 yuan for both the ultimatum and the dictator game. Here we test the general question of whether traces of Chinese socio-economic reforms can be detected in the two experimental games, as defined in Predictions 1 to 6.

**Table 1:** Participants' offers (in percent) in the ultimatum game and the dictator game in the pilot studies.

		Ultimatum game		Dictator game		
		Mean (%)	SE (%)	Mean (%)	SE (%)	N
Payment plan	Outcome-dependent payment	49.0	1.7	37.8	2.8	54
	Outcome-independent payment	46.3	2.0	35.1	2.4	57
Stake (RMB)	20 yuan	46.3	2.0	35.1	2.4	57
	100 yuan	44.4	2.4	31.7	4.0	31

### Methods

#### Participants

Two hundred and fifty-three participants were recruited, including college students, employees in companies, and community residents; 157 were males and 96 females. Because 5 of them did not provide age information, only 248 participants entered the analysis. Cohort I consisted of citizens born before or in 1950 and included citizens who at the time of the study were at least 55 years old, which is the typical retirement age in China. Many of them were recruited among community residents, and most of them were born before 1949, the year New China was established; all experienced socialist reform and the Cultural Revolution in the Mao era. Cohort II consisted of citizens born from 1951 to 1975. Cohort III consisted of citizens born after the Mao era, that is, 1976 and later, who grew up during the new economic reforms.

### Procedure

Participants played the ultimatum game as both proposers and responders, and the dictator game as proposers only. The procedure for the two games was the same as in the pilot studies. In the round of the ultimatum game in which participants were responders, the strategy method [24] was used; that is, participants were asked to write down the minimal offer they would accept.

For the elderly subjects (Cohort I participants), experimenters read the instructions aloud and helped them to understand these and in writing down their answers when necessary. The order of the ultimatum and the dictator game (proposers) was counterbalanced. The ultimatum game in which participants were responders was always the last game. No differences in offers and minimum accepted offers were observed between genders, consistent with Solnick's [25] findings.

### Results

#### Prediction 1: 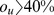 for the oldest cohort

**Figure 1:**
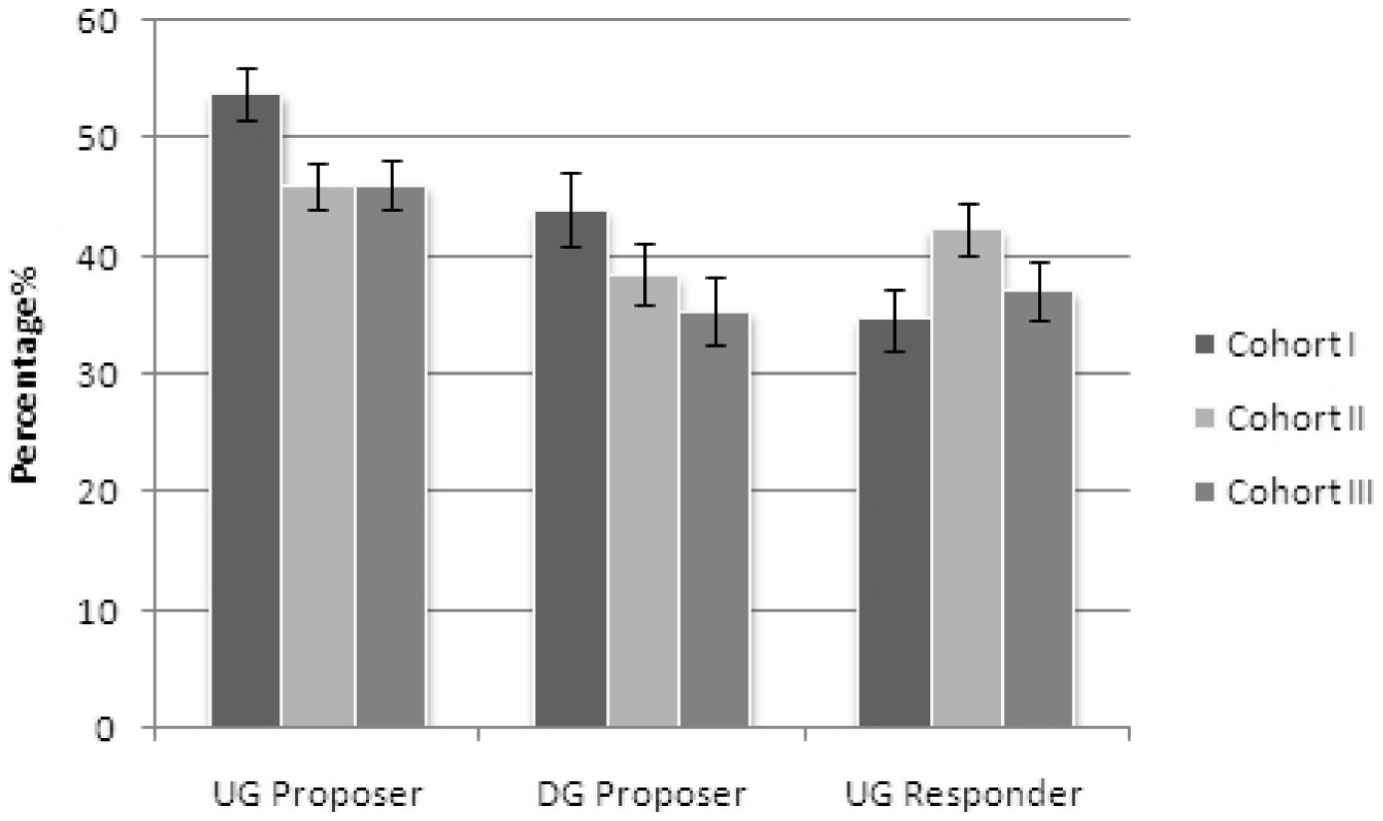
Mean offers (in %) for the ultimatum game (UG) and dictator game (DG) and minimal accepted offer for the ultimatum games. **(The error bar is +/−1 standard error of mean.)**

Table 2 and Figure 1 show that the oldest cohort's mean offer was 53.8% in the ultimatum game. Means above 50% have been rarely observed in the literature, confirming Prediction 1.

**Table 2:** Participants' offers (in % of 100 yuan) in the ultimatum game and the dictator game by cohorts.

	Ultimatum game			Dictator game		
Time of birth (cohorts)	Mea(%)	SE (%)	N	Mean (%)	SE(%)	N
Cohort I: Born ≤1950	53.8	2.3	68	44.0	3.1	67
Cohort II: Born 1951 to 1975	46.0	1.9	95	38.5	2.6	96
Cohort III: Born ≥1976	46.1	2.1	84	35.4	2.8	84

#### Prediction 2: 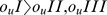


Table 2 shows that in the ultimatum game, Cohort I's mean offer was higher than Cohort II's (*SE* = 3.0; *p* = .03) and Cohort III's (*SE* = 3.1; *p* = .04), consistent with Prediction 2.

#### Prediction 3: 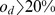 for the oldest cohort

The offers in the dictator game were meant to disentangle high offers in the ultimatum game that were equality-motivated from purely strategic ones. In the oldest group, the mean offer in the dictator game was 44%, consistent with Prediction 3. The difference between the mean offers in the two games (7.8 percentage points) indicates some degree of strategic thinking, yet the absolute value is unusually high for the dictator game.

#### Prediction 4: 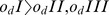


Mean offers were highest for the oldest cohort in the dictator game, consistent with Prediction 4 (Table 2). As the overlap of the standard errors in Figure 1 shows, however, the differences between Cohorts I and II (*SE* = 4.1; *p* = .39) and Cohorts I and III (*SE* = 4.2; *p* = .12) were relatively small compared to the variability in the dictator game offers, which in all three cohorts was larger than that in the ultimatum game.

#### Prediction 5: A positive correlation between age and offer in the middle cohort, for both the ultimatum and the dictator games; no such correlation for the two other cohorts

Consistent with this prediction, correlations were positive only for Cohort II and in both games (Table 3). All other correlations between age and offer were zero or negative in the two other cohorts. Because the correlation for the ultimatum game was quite low, this prediction is only partially confirmed by the data.

**Table 3:** Correlations between age and offer (or minimum accepted offer) for the ultimatum game (UG) and the dictator game (DG).

Cohort	UG offer	DG offer	UG responder	N
Cohort I: Born ≤1950	−0.08 *p* = 0.53	−0.30 *p* = 0.02	0.12 *p* = 0.33	68
Cohort II: Born 1951 to 1975	0.14 *p* = 0.19	0.38 *p* = 0.00	−0.11 *p* = 0.31	96
Cohort III: Born ≥1976	−0.06 *p* = 0.61	−0.18 *p* = 0.11	−0.04 *p* = 0.69	84
All Cohorts	0.16 *p* = 0.01	0.13 *p* = 0.04	−0.05 *p* = 0.42	248

#### Prediction 6: 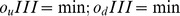


Mean offers in the dictator game were lowest for the post-Mao cohort (Cohort III), and offers in the ultimatum game were about equal for post-Mao and the middle cohort. Thus, the general tendency of Prediction 6 is correct, but the difference is small or nonexistent with respect to the middle cohort,.

#### Mean minimal accepted offers

Table 4 and Figure 1 show that the responders' mean minimal accepted offer tended to vary in an inversely U-shaped way. Both the oldest and the post-Mao cohort accepted smaller offers than the middle group. The overall level was substantially higher for all three groups than levels reported in the literature.

**Table 4:** Participants'minimal accepted offer (in % of 100 yuan) by cohorts.

Time of birth (cohorts)	Mean (%)	SE (%)	N
Cohort I: Born ≤1950	34.7	2.6	68
Cohort II: Born 1951 to 1975	42.4	2.2	94
Cohort III: Born ≥1976	37.1	2.4	84

## Discussion

Radical social and economic change took place in a relatively short period in China: from planned economy to market economy, with social value orientations changing accordingly. We asked whether this change has left its traces in Chinese citizens' social behavior in the ultimatum and dictator games. We defined this general question in terms of a simple model that assumes that the longer a person lived under Mao's rule, the higher his or her offers would be in the two games. This model leads to six specific predictions, tested in a sample of 248 Chinese people.

First, the results showed that Cohort I, in which everyone spent the maximum possible time of about 27 years under Mao's leadership, offered on average 54% in the ultimatum game, which is far higher than the average 40% offer reported in the literature [15]. Second, this cohort offered more than each of the two other cohorts in the ultimatum game. Third, it offered on average 44% in the dictator game, which is far more than the typical mean offer of around 20% reported in the literature [18], [5]. Fourth, Cohort I offered more than each of the two other cohorts in the dictator game, although here the tendency is correctly predicted but the standard deviations partially overlap. Fifth, the model predicts a positive correlation between age and offer only in the middle cohort (Cohort II), because it is the only one in which *t* (years spent under Mao's regime) varies between members and age is a linear function of *t*. Consistent with this prediction, there were positive correlations in Cohort II, both in the ultimatum game (*r* = .19) and dictator game (*r* = .38), and no positive correlations in the oldest cohort and the post-Mao cohort, in each of which all members spent equal time under Mao's regime: the maximum time for the oldest and zero for the youngest. Finally, the post-Mao cohort's mean offers were about equally low as those of the middle cohort in the ultimatum game and the lowest in the dictator game, where strategic considerations are excluded.

To summarize, the general pattern of results is consistent with the hypothesis that traces of the Chinese socio-economic reforms can be detected in people's offers in the ultimatum and dictator games.

### Traces of the socio-economic reforms or age?

The differences between the offers in the three groups may reflect not a cohort effect, however, but a simple age effect. For instance, the observation that the oldest cohort gave mean offers in both the ultimatum and the dictator game close to equality (in the case of the ultimatum game even higher than 50%) might reflect that Chinese citizens become more generous with age. Van Lange, Otten, De Gruin, and Joirenman [26] reported, for instance, that the prevalence of pro-social behavior in Dutch citizens increased with age from early adulthood (15+) to old age (60+), and Engel [19] found that old age correlated with higher offers. These studies, however, did not include a cohort analysis. If the effects reported were due to age, then one should find positive correlations between age and offer *within* each cohort, not only in the middle cohort (as stated in Prediction 5), where age directly reflects the time spent under Mao's regime. As Table 3, however, shows for the ultimatum game, no positive correlations between age and offer exist within Cohorts I and III. The same holds for the dictator game. Positive correlations appear only in the middle cohort, where age directly reflects the time under Mao's regime. Thus, the analysis in Table 3 does not support the alternative hypothesis that people make more generous offers the older they are.

### Limitations and Open Questions

This conclusion, however, needs to be drawn with caution, given some limitations to the present study. First, the selection of participants was based on a convenience sample rather than a representative sample of the Chinese population, the latter being difficult to obtain in China. To achieve some degree of heterogeneity, we therefore drew our samples from a large city, Beijing. Second, all games were played with one of the experimenters rather than with other persons from the cohort, and the payment was outcome-independent. Results from the first pilot study, however, indicated that payment method appears to have little or no influence on offers.

This study also provided unexpected results that call for explanation. In the dictator game, which attempts to measure purely altruistic behavior, the participants in the middle cohort offered larger amounts with increasing age, whereas this correlation was negative (*r* = −.30, *p* = .02) in the oldest group. Why, within the cohort of older citizens, would those who are younger offer more? The proposed model predicts only the positive correlation in the middle cohort, assuming that offers increase with the years an individual was exposed to Mao's regime. It makes no predictions for the oldest cohort, where the number of years of exposure remains constant. In this cohort, being older means that more of one's formative years occurred before Mao's regime. The oldest members of Cohort I were already about 30 years old when Mao's regime began. Thus, the youngest members of the oldest cohort are most comparable to the oldest members of the middle cohort, which may account for the inverted correlation. In other words, not only the total amount of time but also the number of formative years spent during the Mao era may strengthen conformity with Maoist values, increasing those participants' offers in the experimental games.

### The Cultural Transmission of Values

Game theory has been based on an individualistic, if not egoistic and antagonistic view of human rationality. Although philosophers and economists in the United States embraced game theory in the early years of the Cold War, it did not cohere with and was mostly ignored by the human sciences in the former Soviet Union [27]. Widely respected communist moral rules such as equal division and to “not care about oneself but others” [8] were not part of the “axioms” of game theory. But even in Western societies, equality is actually used as a heuristic principle for decision making [28]–[29], including parental investment [30] and moral behavior [31]. While fairness and equal division are sometimes discussed solely as the product of an evolved innate psychology, we focused on a different source of equality, the socio-economic values of the Mao era. We agree with Henrich [32] that economic decisions can be heavily influenced by cultural differences–that is, by socially transmitted rules that may vary from group to group as a consequence of different cultural evolutionary trajectories. Whereas Henrich et al. [4] showed substantial differences between small-scale societies, we analyzed differences within one large-scale society.

We consider the present study as a first attempt to demonstrate that socio-economic changes within the same society, in this case from Mao Zedong's era to the social-economic reforms led by Deng Xiaoping, can explain individual differences in distribution. In China today, equal poverty is no longer a source of pride, as it was under Mao. But people's sense of altruism and collectivism appears to leave its traces in the choices made in experimental games.
